# Retraction: 2-Day versus C-reactive protein guided antibiotherapy with levofloxacin in acute COPD exacerbation: A randomized controlled trial

**DOI:** 10.1371/journal.pone.0297557

**Published:** 2024-01-18

**Authors:** The PLOS ONE Editors

After publication of this article [1, 2], concerns were raised about the relationship of this work to a subsequent article for which an Expression of Concern was published in 2023 [3, 4].

The study published in [1, 2] was not cited and discussed in [3] and the following concerns were noted:

- Studies [1, 2] and [3] have the same number of excluded patients (177).
- Article [1, 2] reports the study was conducted between May 2017 and January 2019, and article [3] reports the study was conducted between February 2018 and January 2021 with patients from three of the same emergency departments and identical inclusion criteria.

In following up on these issues, it was also noted that:

- Changes were made to the registered protocol [6], including the number of study groups, their description, study status, completion date, outcomes, timeframes, and eligibility criteria during and after the reported study period of May 2017 to January 2019 [1, 2].
- There is text overlap between the Statistical Analysis section in the Materials and Methods section of [1, 2] and the Statistical Analysis section in the Methods section in [5].

The corresponding author stated that the overall study design for articles [1, 2] and [3] was to compare four therapeutic strategies to investigate which method can shorten the duration of antibiotic therapy while ensuring the same effectiveness as the standard duration (7 days). These four strategies were: a) 2 days levofloxacin plus levofloxacin with CRP-guided duration, b) 2 days levofloxacin plus placebo with CRP-guided duration, c) 2 days levofloxacin plus placebo 5 days, and d) 7 days levofloxacin. The corresponding author noted that article [1, 2] compares strategies a) and b), and article [3] compares strategies c) and d).

The corresponding author also stated that for each individual participating center, recruitment for the second study [3] was completed after the recruitment period for [1, 2] was closed at the end of the first study [1, 2], but that one of the participating centers started recruitment for the second study [3] whilst the other participating centers were still completing the recruitment period for [1, 2]. They stated that similarities in the sample sizes between groups were planned but that the two studies did not include any overlap in the participants, and that the demographic and clinical characteristics on admission were different. The corresponding author stated that similarities in these would be expected because the groups were recruited from the same overall population. Regarding the same number of patients (n = 177) excluded for not meeting inclusion criteria in articles [1, 2] and [3], the corresponding and fourth authors stated that this is a coincidence.

The fourth author confirmed that changes were made to the registered trial protocol at multiple timepoints, and stated that these changes were made due to the evolving needs of the study, there was a delay in reporting these changes in clinicaltrials.gov compared to when they were implemented, and the changes were approved by the ethics committee before they were implemented. They did not clarify the lengths of the delays in reporting the registered trial protocol changes in clinicaltrials.gov compared to when they were implemented. Ethical approval documents covering all institutions, all amendments, and the original study protocol were not provided in full and the *PLOS ONE* Editors have concerns about the authenticity of some of the documents provided.

In light of the unresolved questions about the datasets, randomization, changes to the registered trial protocol, and whether the study met with appropriate clinical ethical standards, the *PLOS ONE* Editors retract this article [1, 2].

BHAK, GMH, MMA, KMH, AA, BH, RR, TI, BBN, BR, BW, MS, NS, BDY, and BA did not agree with the retraction. SA, BK, and MM either did not respond directly or could not be reached.

## References

[1] Mohamed Amine M, Selma M, Adel S, Khaoula Bha, Mohamed Hassene K, Imen, et al. (2021) 2-Day versus C-reactive protein guided antibiotherapy with levofloxacin in acute COPD exacerbation: A randomized controlled trial. PLoS ONE 16(5): e0251716. 10.1371/journal.pone.0251716 34015041 PMC8136675

[2] The *PLOS ONE* Staff (2021) Correction: 2-Day versus C-reactive protein guided antibiotherapy with levofloxacin in acute COPD exacerbation: A randomized controlled trial. PLoS ONE 16(6): e0253252. 10.1371/journal.pone.0253252 34106990 PMC8189478

[3] Messous S, Trabelsi I, Bel Haj Ali K, et al. (2022) Two-day versus seven-day course of levofloxacin in acute COPD exacerbation: a randomized controlled trial. *Therapeutic Advances in Respiratory Disease*. 16. doi: 10.1177/17534666221099729 35657073 PMC9168850

[4] (2023) Expression of concern: ‘Two-day versus seven-day course of levofloxacin in acute COPD exacerbation: a randomized controlled trial’. *Therapeutic Advances in Respiratory Disease*. 17. doi: 10.1177/17534666231190750 37571965 PMC10423443

[5] Verduri A, Luppi F, D’Amico R, Balduzzi S, Vicini R, Liverani A, et al. (2015) Antibiotic Treatment of Severe Exacerbations of Chronic Obstructive Pulmonary Disease with Procalcitonin: A Randomized Noninferiority Trial. PLoS ONE 10(3): e0118241. doi: 10.1371/journal.pone.0118241 25760346 PMC4356612

[6] https://clinicaltrials.gov/study/NCT02067780

